# Association between patellar tendon moment arm and running performance in endurance runners

**DOI:** 10.14814/phy2.14981

**Published:** 2021-08-02

**Authors:** Hiromasa Ueno, Tadashi Suga, Kenji Takao, Takahiro Tanaka, Yuto Miyake, Yuki Kusagawa, Masafumi Terada, Akinori Nagano, Tadao Isaka

**Affiliations:** ^1^ Faculty of Sport and Health Science Ritsumeikan University Shiga Japan; ^2^ Graduate School of Health and Sport Science Nippon Sport Science University Tokyo Japan; ^3^ Japan Society for the Promotion of Science Tokyo Japan

**Keywords:** joint torque, magnetic resonance imaging, muscle volume, quadriceps femoris

## Abstract

A shorter joint moment arm (MA) may help maintain the necessary muscle force when muscle contractions are repeated. This beneficial effect may contribute to reducing the energy cost during running. In this study, we examined the correlation between patellar tendon MA and running performance in endurance runners. The patellar tendon MA and quadriceps femoris muscle volume (MV) in 42 male endurance runners and 14 body size‐matched male untrained participants were measured using a 1.5‐T magnetic resonance system. The patellar tendon MA was significantly shorter in endurance runners than in untrained participants (*p* = 0.034, *d* = 0.65). In endurance runners, shorter patellar tendon MA correlated significantly with better personal best 5000‐m race rime (*r* = 0.322, *p* = 0.034). A trend toward such a significant correlation was obtained between quadriceps femoris MV and personal best 5000‐m race time (*r* = 0.303, *p* = 0.051). Although the correlation between patellar tendon MA and personal best 5000‐m race time did not remain significant after adjusting for the quadriceps femoris MV (partial *r* = 0.247, *p* = 0.120), a stepwise multiple regression analysis (conducted with body height, body mass, patellar tendon MA, and quadriceps femoris MV) selected the patellar tendon MA (*β* = 0.322) as only a predictive variable for the personal best 5000‐m race time (adjusted *R* ^2^ = 0.081, *p* = 0.038). These findings suggest that the shorter patellar tendon MA, partially accorded with the smaller quadriceps femoris size, may be a favorable morphological variable for better running performance in endurance runners.

## Graphical abstract

This study examined the relationship between patellar tendon moment arm (MA) and running performance in endurance runners. A shorter patellar tendon MA was correlated with a better personal best 5000‐m race rime in endurance runners. The shorter patellar tendon MA may be a favorable morphological variable for superior running performance in endurance runners.

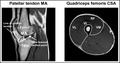

## 1 INTRODUCTION

In general, an increase in the running velocity is associated with increasing the torques produced from the lower limb joints (i.e., the hip, knee, and ankle joints) during running (Arampatzis et al., 1999; Belli et al., 2002). Of these joints, we and others have previously reported that the favorable morphological variables related to the torque production of the ankle joint (e.g., the Achilles tendon length, Achilles tendon moment arm dimension, and forefoot bone length) may be associated with better running performance in endurance runners (Barnes et al., 2014; Hunter et al., 2011; Scholz et al., 2008; Ueno et al., 2018a, 2018b). Unlike the relationships on the ankle joint torque‐related morphological variables, the effect of the knee joint torque‐related morphological variable(s) on running performance is poorly understood. The knee extensor torque is mainly required to obtaining propulsion force during the contact phase and to swinging the legs rapidly during the swing phase while running (Kenneally‐Dabrowski et al., 2019; Novacheck, 1998). Indeed, an increase in the running velocity is partially associated with increasing the knee extensor torque during running (Arampatzis et al., 1999; Belli et al., 2002). Therefore, the morphological variable(s) related to the knee extensor torque production during running may be important in achieving superior running performance in endurance runners.

The joint torque is generally expressed as the product of the moment arm (MA) and muscle force. Hence, a longer joint MA can potentially increase the joint torque. We and others have previously reported a positive correlation between MA and torque in several joints. (Baxter & Piazza, 2014; Blazevich et al., 2009; Hori et al., 2020; Sugisaki et al., 2010; Tottori et al., 2020). Of these studies, we have demonstrated that the patellar tendon MA (i.e., the distance between the tibio‐femoral contact point and the mid‐line of the patellar tendon), which is calculated as an alternative to the overall knee extensor muscle MA, is correlated with the knee extensor isometric and isokinetic torques in untrained individuals (Hori et al., 2020; Tottori et al., 2020). Therefore, the longer patellar tendon MA may be a favorable morphological variable for achieving superior performance in athletes who participate in sports that require greater knee extensor torque.

The joint MA dimension is potentially determined by agonist muscle size (Vigotsky et al., 2015). Our previous studies reported a positive correlation between patellar tendon MA and quadriceps femoris muscle size (i.e., cross‐sectional area [CSA] and muscle volume [MV]) in untrained individuals (Hori et al., 2020; Miyake et al., 2017; Tomita et al., 2018; Tottori et al., 2020). However, we found the absence of such a correlation in sprinters. Moreover, we reported that although the quadriceps femoris muscle size did not differ between sprinters and body size‐matched untrained participants, the patellar tendon MA was longer in sprinters than in untrained participants (Miyake et al., 2017). Furthermore, we reported that the longer patellar tendon MA is correlated with better sprint performance parameters (i.e., persona best 100‐m race time and 50‐m sprint velocity) independent of the quadriceps femoris size in sprinters (Miyake et al., 2017), which may be potentially due to enhanced knee extensor torque‐producing capacity (i.e., the knee extensor torque per the unit of the quadriceps femoris size: Hori et al., 2020; Tottori et al., 2020). Therefore, the longer patellar tendon MA may be an important morphological variable for achieving better sprint performance in sprinters.

In contrast to our findings in sprinters, several previous studies have reported that in endurance runners, a shorter Achilles tendon MA (i.e., an alternative to the overall plantar flexor muscle MA) may be associated with better running economy (Barnes et al., 2014; Scholz et al., 2008), which is a well‐known important indicator of the running performance (Saunders et al., 2004). Generally, change in the muscle length is expressed as the product of the changes in the joint angle and MA dimension (Spoor & van Leeuwen, 1992). Hence, the favorable effect of the shorter joint MA on running performance may be because this morphology can mitigate the degree of muscle length changes during joint rotation, potentially by decreasing the velocities of muscle shortening and lengthening (Lee & Piazza, 2009; Nagano & Komura, 2003). Such muscle performances secondary to the shorter joint MA may contribute to maintaining the necessary muscle force during repeated muscle contractions, and thus, this may result in improving running economy due to reduced energy consumption in the working muscles during running (Fletcher & MacIntosh, 2017). Therefore, in addition to the Achilles tendon MA, the patellar tendon MA may be an important morphological variable associated with economical running, thereby contributing to better running performance (i.e., personal best time) in endurance runners. Based on this biomechanical background, we hypothesized that the shorter patellar tendon MA would be correlated with better running performance in endurance runners.

Endurance runners are required to having leaner body segments than those of many athletes to reduce the energy cost during running (O'Connor et al., 2007; Pollock et al., 1977; Weyand & Davis, 2005). In particular, the leg muscle mass may affect the energy cost during running, especially during swinging of the legs (Browning et al., 2007; Myers & Steudel, 1985), and thus, the smaller leg muscle size may be useful in achieving superior running performance (Black et al., 2020; Lucia et al., 2006; Scholz et al., 2008), as they provide better running economy owing to lesser leg moments of inertia (Black et al., 2020). In addition, although our previous study reported the absence of a correlation between patellar tendon MA and quadriceps femoris size in sprinters (Miyake et al., 2017), the quadriceps femoris size may be an important determinant for the patellar tendon MA in endurance runners, similar to untrained participants (Hori et al., 2020; Miyake et al., 2017; Tomita et al., 2018; Tottori et al., 2020). Therefore, we also hypothesized that, if shorter patellar tendon MA is correlated with better running performance, it may involve a correlation between smaller quadriceps femoris size and better running performance in endurance runners.

To test our hypotheses, we first compared the patellar tendon MA and quadriceps femoris MV between endurance runners and untrained participants in order to understand the characteristics of these knee extensor morphological variables for endurance runners. Thereafter, we examined the correlations of the patellar tendon MA and quadriceps femoris MV with running performance in endurance runners.

## 2 METHODS

### 2.1 Participants

Prior to the present study, we calculated *a priori* sample size using the effect sizes obtained in our previous studies (Miyake et al., 2017; Tottori et al., 2021). To compare the morphological variable (e.g., muscle size) between two groups, the necessary minimum numbers of participants for each group were 9, which was calculated from an effect size of 1.41 (gluteus maximum CSA: Tottori et al., 2021), α‐level of 0.05, and β‐level of 0.20 (80% power). Additionally, to determine the correlation between morphological variable (e.g., joint MA) and performance in a single athlete group, the necessary minimum number of participants was 18, as calculated from an effect size of 0.61 (patellar tendon MA: Miyake et al., 2017), α‐level of 0.05, and β‐level of 0.2 (80% power).

Forty‐two male endurance runners (age: 20 ± 1 years) and 14 untrained participants (age: 22 ± 1 years) participated in this study. The numbers for each group were sufficient for ensuring statistical power and sensitivity based on the *priori* sample size calculations. The endurance runners were all well‐trained, being involved in regular training and competition. The official personal best times of a 5000‐m race (i.e., personal best 5000‐m race time) in the endurance runners ranged from 13 min 54 s to 15 min 54 (mean, 14 min 53 ± 25 s). A mean weekly training distance in the endurance runners was 112 ± 28 km. The untrained participants whose physical characteristics (i.e., body height and body mass) were similar to those of the endurance runners were recruited as a control group. All participants were informed of the experimental procedures and provided written consent to participate in the study. This study was approved by the Ethics Committee of Ritsumeikan University (BKC‐IRB‐2016‐047) and conducted according to the Declaration of Helsinki.

### 2.2 Magnetic resonance imaging (MRI)

**FIGURE 1:**
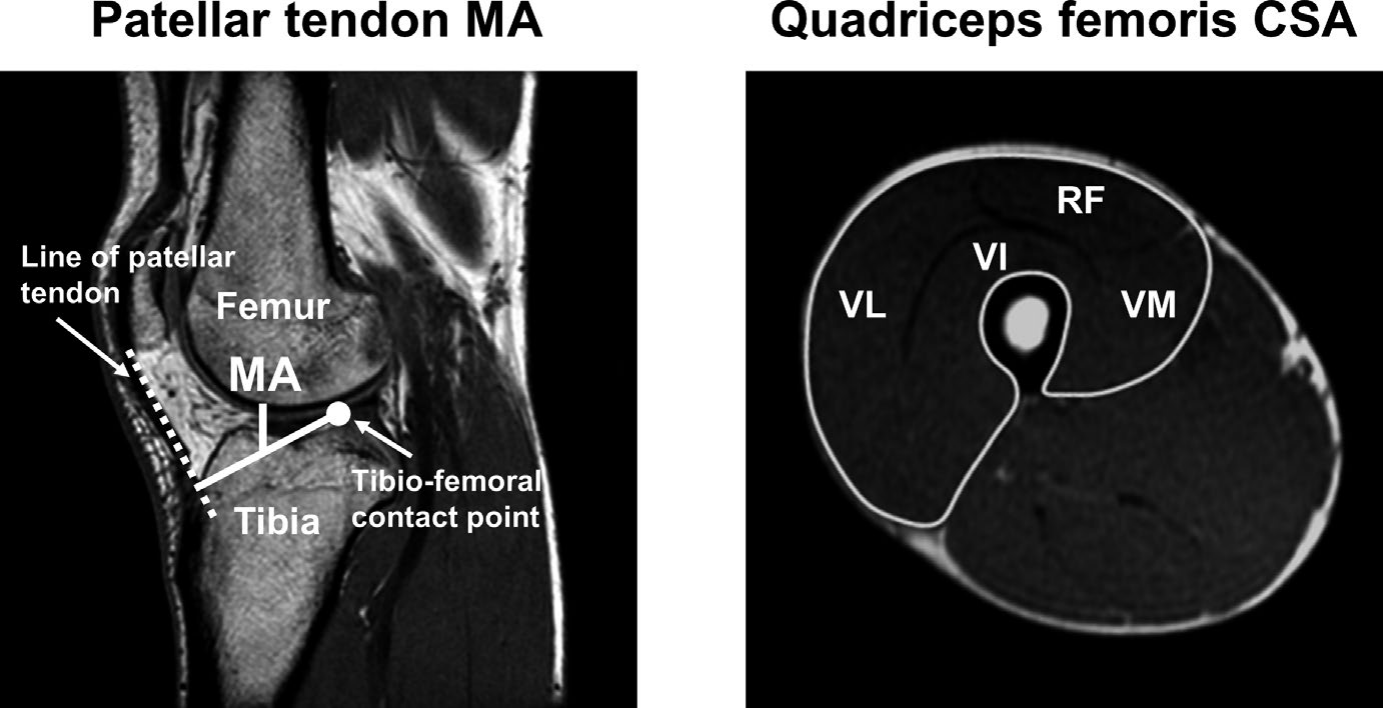
Representative magnetic resonance imaging scans for measuring the patellar tendon moment arm (MA) and quadriceps femoris muscle volume (MV). The left scan shows a sagittal image on the knee joint of the right leg. The patellar tendon MA was calculated as the distance between tibio‐femoral contact point and mid‐line of the patellar tendon. The right scan shows an axial image on the midthigh level of the right thigh. The cross‐sectional areas (CSA) of the quadriceps femoris included the rectus femoris (RF), vastus intermedius (VI), vastus lateralis (VL), and vastus medialis (VM). The quadriceps femoris MV was calculated by multiplying the sum of the CSAs along their length at intervals of 1 cm

Representative MRI scans for measuring the patellar tendon MA and quadriceps femoris MV are shown in Figure 1. These MRI measurements were performed using a 1.5‐T magnetic resonance system (Signa HDxt; GE Medical Systems). To measure the patellar tendon MA and quadriceps femoris MV, participants were placed in a supine position on the scanner bed, with both knees fully extended and both ankles set at the neutral position (i.e., 0°). The participants were also instructed to maintain a relaxed state throughout the MRI measurements.

With regard to the patellar tendon MA measurement, three‐dimensional isotropic T1‐weighted MRI scans of the right knee joint were acquired with an eight channels coil. Sagittal scans were obtained in successive slices with an inter distance of 10 mm with a repetition time of 11.3 ms, echo time of 5.1 ms, slice thickness of 1.2 mm, field of view of 280 mm, and matrix size of 256 × 256 pixels. The patellar tendon MA was calculated as the distance between the tibio‐femoral contact point and the mid‐line of the patellar tendon, as in our and other previous studies (Blazevich et al., 2009; Hori et al., 2020; Miyake et al., 2017; Tomita et al., 2018; Tottori et al., 2020). The measurements of the patellar tendon MA were performed twice, and the mean of the two values was used for the analysis of this study. The coefficient of variations of the two measurements in the patellar tendon MA was 0.5 ± 0.4%. The intraclass correlation coefficient of the two measurements was 0.991.

With regard to the quadriceps femoris MV measurement, axial T1‐weighted MRI scans of the right thigh were acquired with a standard body coil. Axial scans were obtained in successive slices with an inter distance of 10 mm from the inferior aspect of the greater trochanter to the lower edge of the femur with a repetition time of 600 ms, echo time of 7.6 ms, field of view of 480 mm, and matrix size of 512 × 256 pixels. From these scans, CSAs on each slice were measured. The measured quadriceps femoris CSAs involved the rectus femoris, vastus intermedius, vastus lateralis, and vastus medialis. The quadriceps femoris MV was calculated by multiplying the sum of the CSAs along their length at intervals of 1 cm, as in our previous studies (Hori et al., 2020; Tomita et al., 2018; Tottori et al., 2020).

The analyses for the patellar tendon MA and quadriceps femoris MV were conducted using image analysis software (OsiriX Version 5.6). The reproducibility of the patellar tendon MA and quadriceps femoris size on two separate days has been reported in our previous studies (Miyake et al., 2017; Tomita et al., 2018; Tottori et al., 2020).

### 2.3 Statistical analysis

All data are presented as mean ± standard deviation. Comparisons of physical characteristics (i.e., body height and body mass) and knee extensor morphological variables (i.e., patellar tendon MA and quadriceps femoris MV) between endurance runners and untrained participants were performed using an unpaired *t*‐test. The Cohen's *d* effect size using the pooled stranded deviation was calculated to determine the magnitude of differences in the variable between the two groups. The effect size was interpreted as small (0.20–0.49), medium (0.50–0.79), and large (>0.80) (Cohen, 1992). Correlations between knee extensor morphological variables and personal best 5000‐m race time in endurance runners were evaluated using the Pearson's product‐moment correlation coefficient. The strength of a correlation between two variables was interpreted as trivial (<0.10), small (0.10–0.29), medium (0.30–0.49), and large (>0.50) (Cohen, 1992). A partial correlation analysis was used to adjust the effect of the quadriceps femoris MV on a correlation between patellar tendon MA and personal best 5000‐m race time in endurance runners. A stepwise multiple regression analysis was used to determine the predictive variable for the personal best 5000‐m race time in endurance runners. This analysis was conducted with four variables, body height, body mass, patellar tendon MA, and quadriceps femoris MV. The statistical significance level was defined at *p* < 0.05. All statistical analyses were conducted using IBM SPSS software (version 19.0; International Business Machines Corp).

## 3 RESULTS

Body height and body mass did not differ significantly between endurance runners and untrained participants (170.6 ± 6.3 and 171.5 ± 6.5 cm, respectively, *p* = 0.653, *d* = 0.14 for body height: 56.2 ± 5.3 and 58.4 ± 4.5 kg, respectively, *p* = 0.168, *d* = 0.43 for body mass).

**FIGURE 2:**
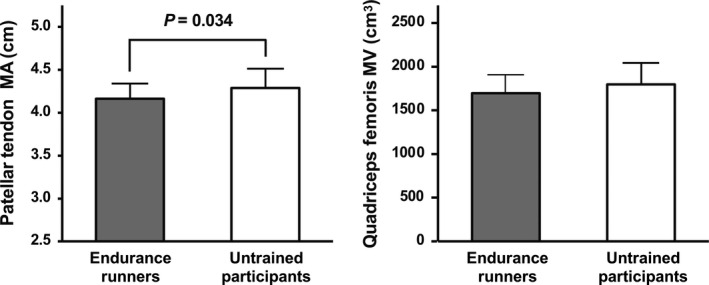
Comparisons of the patellar tendon MA and quadriceps femoris MV between endurance runners and untrained participants. Values are presented as mean ± standard deviation

Comparisons of the patellar tendon MA and quadriceps femoris MV between endurance runners and untrained participants are presented in Figure 2. The patellar tendon MA was significantly shorter in endurance runners than in untrained participants (*p* = 0.034, *d* = 0.65). In contrast, the quadriceps MV did not differ significantly between the two groups (*p* = 0.145, *d* = 0.45).

**FIGURE 3:**
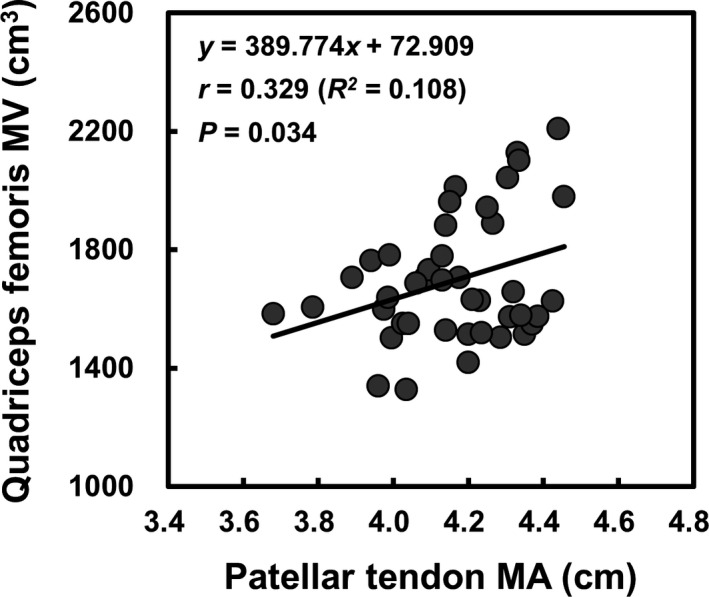
Correlation between patellar MA and quadriceps femoris MV in endurance runners

A correlation between patellar tendon MA and quadriceps femoris MV in endurance runners is presented in Figure 3. The patellar tendon MA correlated significantly with the quadriceps femoris MV in endurance runners (*r* = 0.329, *R* ^2^ = 0.108, *p* = 0.034,). A trend toward such a significant correlation was also observed in untrained participants (*r* = 0.501, *R* ^2^ = 0.251, *p* = 0.068).

**FIGURE 4:**
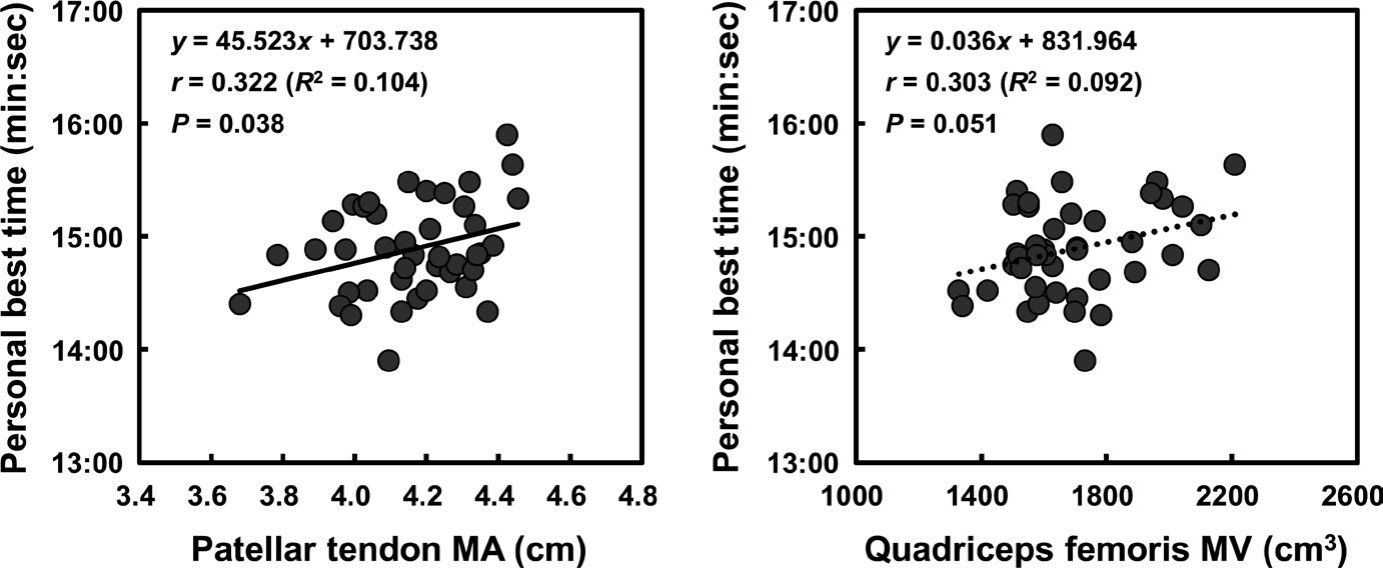
Correlations of patellar tendon MA and quadriceps femoris MV with personal best 5000‐m race time in endurance runners

Correlations of patellar tendon MA and quadriceps femoris MV with personal best 5000‐m race time in endurance runners are presented in Figure 4. The patellar tendon MA correlated significantly with personal best 5000‐m race time (*r* = 0.322, *R* ^2^ = 0.104, *p* = 0.038,). A trend toward such a significant correlation was also observed between quadriceps femoris MV and personal best 5000‐m race time (*r* = 0.303, *R* ^2^ = 0.092, *p* = 0.051).

Although a partial correlation analysis revealed that the correlation between patellar tendon MA and personal best 5000‐m race time did not remain significant after adjusting for the quadriceps femoris MV (partial *r* = 0.247, *R* ^2^ = 0.061, *p* = 0.120), a stepwise multiple regression analysis revealed that the patellar tendon MA (*β* = 0.322) was the only predictive variable of the personal best 5000‐m race time (*r* = 0.322, *R* ^2^ = 0.104, adjusted *R* ^2^ = 0.322, *p* = 0.038).

## 4 DISCUSSION

In this study, we demonstrated the correlation between shorter patellar tendon MA and better personal best 5000‐m race time in endurance runners. Several previous studies have reported that shorter Achilles tendon MA is correlated with better running performance in endurance runners (Barnes et al., 2014; Scholz et al., 2008). However, no study has examined the correlation between patellar tendon MA and running performance prior to this study. Therefore, the preset study is the first to determine this correlation by showing that the shorter patellar tendon MA may be a favorable morphological variable for better running performance in endurance runners.

The shorter joint MA can mitigate the length and velocity changes in the working muscles during joint rotation (Lee & Piazza, 2009; Nagano & Komura, 2003). Hence, the shorter patellar tendon MA may contribute to reductions in the contractile energy cost and energy consumption in the knee extensor muscles (i.e., the quadriceps femoris) during running (Fletcher & MacIntosh, 2017). These favorable muscle performances secondary to the shorter patellar tendon MA may result in improving running performance. Additionally, the shorter patellar tendon MA can decrease the radius of gyration of knee joint rotation. Thus, this morphology may facilitate faster joint rotation, thereby allowing for a rapid knee extension, particularly during swinging of the legs in the swing phase while running. The rapid knee extension may help achieve faster running velocity, potentially by shortening the contact and swing times during running (Kenneally‐Dabrowski et al., 2019; Novacheck, 1998). These shortened contact and swing times contribute to an increased step frequency (Kenneally‐Dabrowski et al., 2019), which is useful in improving the running performance (Barnes & Kilding, 2015; Quinn et al., 2019). Therefore, runners with shorter patellar tendon MA can achieve superior running performance compared to those with longer patellar tendon MA, potentially due to better economical running arising from biomechanical advantages of the muscular and kinematic performances.

In this study, we identified a correlation tendency between smaller quadriceps femoris MV and better personal best 5000‐m race time in endurance runners. A small mass of the legs may contribute to an improved running economy (Black et al., 2020; Lucia et al., 2006; Scholz et al., 2008), potentially due to decreased leg moments of inertia (Black et al., 2020). Thus, smaller quadriceps femoris size (i.e., MV) may at least partially contribute to improving running performance. Several previous studies have reported that the smaller lower leg mass is correlated with a better running economy in endurance runners (Black et al., 2020; Lucia et al., 2006; Scholz et al., 2008). Furthermore, only one study by Black et al., (2020) demonstrated that the smaller thigh mass, measured using dual‐energy x‐ray absorptiometry, is correlated with a better running economy in both male and female endurance runners (*r* = 0.644 and 0.747, respectively). However, to the best of our knowledge, no study has examined the correlation between specific thigh muscle size and running performance in endurance runners prior to this study. Therefore, the preset study is also the first to determine this correlation by showing that the smaller quadriceps femoris MV may be a favorable morphological variable for better running performance in endurance runners.

This study showed that the shorter patellar tendon MA is significantly correlated with better personal best 5000‐m race time in endurance runners. However, this correlation did not remain significant after adjusting for the quadriceps femoris MV. Additionally, a stepwise multiple regression analysis revealed that the patellar tendon MA was the only predictive variable of the personal best 5000‐m race time, suggesting that it is a more important morphological variable than the quadriceps femoris MV. Nevertheless, in an additional analysis conducted in this study, a correlation between the measured and predicted personal best 5000‐m race times examined using an equation modeled by the stepwise multiple regression analysis was relatively low when compared with that examined using an equation modeled by a multiple regression analysis with the forced entry procedure (adjusted *R* ^2^ = 0.104, *p* = 0.045) that incorporated both patellar tendon MA and quadriceps femoris MV (*r* = 0.322 and 0.384, respectively). Therefore, these findings suggest that the correlation between smaller patellar tendon MA and running performance in endurance runners may be mediated by their smaller quadriceps femoris size.

We and others have previously reported a positive correlation between joint MA and muscle size in untrained individuals (Baxter & Piazza, 2014; Hori et al., 2020; Miyake et al., 2017; Sugisaki et al., 2010; Tomita et al., 2018; Tottori et al., 2020). In this study, we also determined a similar correlation in endurance runners. Our findings indicate that the interaction may exist between patellar tendon MA and quadriceps femoris size in endurance runners as well as untrained participants. Additionally, we found that the patellar tendon MA was shorter in endurance runners than in body size‐matched untrained participants, despite the lack of a difference in the quadriceps femoris MV between the two groups. These present findings suggest that endurance runners may be specifically modeled with smaller patellar tendon MA, which may be attributed to the fact that athletes with smaller patellar tendon MA may selectively participate in long‐distance events, potentially due to the advantage on running performance conferred by the favorable morphology.

In contrast to the findings in endurance runners obtained in this study, we have previously reported that the patellar tendon MA is longer in sprinters than in body size‐matched untrained participants (Miyake et al., 2017), despite the lack of a difference in the quadriceps femoris size (i.e., CSA) between the two groups. Furthermore, our previous studies found the absence of a correlation between patellar tendon MA and quadriceps femoris size in sprinters (Miyake et al., 2017; Tomita et al., 2018). These findings suggest that sprinters may be specifically modeled with longer patellar tendon MA, independent of the quadriceps femoris size. The patellar tendon MA dimension is partially determined by the sagittal length of the distal end of the femur and patellofemoral cartilage thickness. Gratzke et al., (2007) reported that the patellofemoral cartilage thickness was larger in professional power sport athletes (i.e., weight lifters and bobsled sprinters) than in untrained participants. Their result may at least partially contribute to our understanding of having the longer patellar tendon MA in sprinters. Nevertheless, the effects of long‐term training on the femur and patellofemoral cartilage formations in endurance runners and sprinters remain unknown. In general, the magnitude of mechanical stress exposed to the joint appears to affect the bone and cartilage formations (Eckstein et al., 2006; Huiskes et al., 2000), by mediating the expression of the growth factors related to the growth and development of these tissues (Huang et al., 2018; Thielen et al., 2019). Because of a smaller mechanical stress intensity on the knee joint during running than sprinting (Belli et al., 2002), long‐term running training may not significantly influence the femur and patellofemoral cartilage formations in endurance runners.

The findings of our studies identified that shorter patellar tendon MA is a specific characteristic of endurance runners, whereas longer patellar tendon MA is a specific characteristic of sprinters. Furthermore, the shorter patellar tendon MA is advantageous for better running performance in endurance runners, whereas the longer patellar tendon MA is advantageous for better sprint performance in sprinters. Therefore, in the clinical setting, measurement of the patellar tendon MA may be useful for selecting athletic events and for understanding the individual features in athletes, including endurance runners and sprinters.

Recent reviews have suggested that high‐intensity resistance training improves running economy, potentially by ameliorating the mechanical properties (e.g., stiffness) of the muscle‐tendon unit, which is related to the ability of elastic energy utilization during running (Alcaraz‐Ibañez & Rodríguez‐Pérez, 2018; Blagrove et al., 2018). A potential correlation between smaller quadriceps femoris MV and better running performance in endurance runners obtained in this study indicates that excessive hypertrophy of the quadriceps femoris induced by high‐intensity resistance training may reduce their running performance. Therefore, in the clinical setting, measurement of the quadriceps femoris size may be useful for creating and identifying effective training programs to improve running performance for individual endurance runners.

The present study has several limitations. First, in this study, we recruited 42 endurance runners and 14 untrained participants; thus, both groups had a different number of participants. Because the body mass is generally heavier in untrained individuals than in endurance runners (O'Connor et al., 2007), we could not recruit a similar number of body size (especially body mass)‐matched untrained participants to the number of endurance runners. Considering an appropriate statistical approach, with similar sample sizes between the groups, further studies are needed to reexamine the differences in the knee extensor morphological variables between endurance runners and untrained participants. Second, based on the results of this study, we speculated that the correlation between shorter patellar tendon MA and better running performance in endurance runners may be at least partially attributed to the fact that shorter patellar tendon MA may favorably change the contractile performance (e.g., length and velocity changes) of the knee extensor muscles while running. In addition to the joint MA, the tendon length can also change the muscle contractile performances, particularly with contractile velocity (Ishikawa et al., 2007). Hunter et al., (2011) reported that a longer patellar tendon is correlated with better running economy in endurance runners. Furthermore, the muscle fascicle length is known to be a determinant of the muscle shortening velocity (Kumagai et al., 2000); thus, shorter fascicles of the knee extensor muscles may contribute to better running performance. To deepen the present findings, further studies are needed to examine the correlation between patellar tendon MA and contractile performance of the knee extensor muscles while running. Additionally, the interactions between patellar tendon MA and other morphological variables (e.g., patellar tendon length and quadriceps femoris muscle fascicle length) that contribute to the knee extensor muscle contractile performances should be investigated. Third, although this study demonstrated potential correlations of shorter patellar tendon MA and smaller quadriceps femoris MV with better personal best 5000‐m race time in endurance runners (i.e., *r* = 0.322 and 0.303, respectively), these correlations are interpreted as medium (Cohen, 1992). Furthermore, based on the coefficients of determination (i.e., *R* ^2^ = 0.104 and 0.092, respectively) of these correlations, the patellar tendon MA and quadriceps femoris MV were explained by only about 10% of the variance of the personal best 5000‐m race time. A similar trend was also observed in the stepwise multiple regression analysis (i.e., adjusted *R* ^2^ = 0.081). The running performance is known to be determined by various factors (Saunders et al., 2004), including morphological factors. We and others have previously reported that morphological variables pertaining to the ankle joint (e.g., the Achilles tendon length, Achilles tendon MA dimension, and forefoot bone length) are correlated with running performance in endurance runners (Barnes et al., 2014; Hunter et al., 2011; Scholz et al., 2008; Ueno et al., 2018a, 2018b). To clarify the underlying effects of the knee extensor morphological variables on running performance, further studies are needed to examine the correlations of the morphological variables of both ankle and knee joints with running performance in endurance runners.

## 5 CONCLUSION

This study found that a shorter patellar tendon MA is correlated with better running performance in endurance runners. This study also identified a correlation tendency between smaller quadriceps MV and better running performance in endurance runners. Therefore, we suggest that the shorter patellar tendon MA, partially accorded with the smaller quadriceps femoris size, may be a favorable morphological variable for superior running performance in endurance runners.

## CONFLICT OF INTEREST

The authors declare that they have no conflict of interest.

## AUTHOR CONTRIBUTIONS

HU and TS conceived and designed the study; HU, TS, KT, TT, YM, YK, and MT performed the experiments; HU and TS analyzed the data; HU, TS, KT, TT, YM, YK, MT, AN, and TI interpreted the results of the experiments; HU and TS wrote the manuscript; TS, MT, AN, and TI edited and revised the manuscript. All authors have read and approved the manuscript.
